# Assessment of Reliability, Agreement, and Accuracy of Masseter Muscle Ultrasound Thickness Measurement Using a New Standardized Protocol

**DOI:** 10.3390/diagnostics14161771

**Published:** 2024-08-14

**Authors:** Mateusz Rogulski, Małgorzata Pałac, Tomasz Wolny, Paweł Linek

**Affiliations:** 1 Musculoskeletal Diagnostic and Physiotherapy—Research Team, The Jerzy Kukuczka Academy of Physical Education, 40-065 Katowice, Poland; rogulski21@interia.pl (M.R.); malgorzatapalac3@gmail.com (M.P.); 2 Musculoskeletal Elastography and Ultrasonography Laboratory, Institute of Physiotherapy and Health Sciences, The Jerzy Kukuczka Academy of Physical Education, 40-065 Katowice, Poland; t.wolny@twreha.com

**Keywords:** masseter muscle, ultrasound imaging, reliability, computed tomography

## Abstract

There is no validated method of assessing masseter muscle thickness (MMT) by ultrasound imaging (US). However, this is important to ensure study and measurement quality of MMT by US in future studies, as MMT differs depending on the examined area. Thus, this study’s aim was to present a new standardized method for assessing the MMT by US and to evaluate the reliability, consistency, and accuracy of its measurements. We also compared the results of MMT measurements obtained by US and computer tomography (CT). The study included nine healthy adults. The US and CT scans were collected in a supine rest position with the mandible in relaxed position. US measurements were determined according to a new standardized protocol (with precise probe location). The MMT measured by CT and US over a seven-day interval showed excellent intra-rater reliability. The mean MMT measured by CT was 12.1 mm (1.74) on the right side and 11.9 mm (1.61) on the left side. The mean MMT measured by US was 12.7 mm (2.00) on the right side and 11.5 mm (1.37) on the left side. The mean percent error in MMT measurement between CT and US was below 6%. A strong linear relationship was found between the CT and US measurements of the MMT on both body sides (*p* < 0.001, r ≥ 0.93). The proposed method of MMT measurement using US demonstrated excellent reliability, yielding results similar to those obtained from CT images. We recommend the use of this standardization protocol in further studies where precise assessment of MMT by US is expected.

## 1. Introduction

The masseter muscle is one of the muscles of mastication, playing a crucial role in the physiological movements and positioning of the mandible. It has also been associated with temporomandibular joint (TMJ) disorders, craniomandibular dysfunctions, bruxism, and orofacial pain [1,2,3]. Masseter muscle thickness is a parameter commonly analyzed in such studies, and is linked to certain features of the dental arches [4,5], anthropometry [6], and dental disorders [7]. The thickness of the masseter muscle has also been considered in the context of systemic diseases such as sarcopenia and osteoporosis [8,9,10,11,12,13,14,15,16,17,18].

Masseter muscle thickness is generally assessed using ultrasound imaging (US), a non-invasive, cost-effective procedure widely available for use in both children and adults [19]. More complex diagnostic tools such as computed tomography (CT) and magnetic resonance imaging (MRI) are also employed to assess the thickness of the masseter muscle. Although CT and MRI scans provide accurate and reliable cross-sectional images of muscles, including the masseter, they are not routinely used due to their variable availability and relatively high costs [20].

Recently, Gawriołek et al. [21] confirmed that masseter muscle thickness significantly differs depending on the area examined, and strongly suggested the necessity of examining the masseter muscle in specified areas with both coronal and axial projections to achieve objective and repeatable results. To the best of our knowledge, no standardized and precisely validated method for assessing masseter muscle thickness using US currently exists in the literature [7,20,22,23,24,25]. Blicharz et al. [26] reviewed various methods for measuring and assessing masseter muscle thickness using US, highlighting that none of these procedures have been fully verified in terms of reliability and accuracy. Given the important role of the masseter muscle in clinical and research settings, there is a clear need for standardization in US measurement of its thickness. The absence of such standardization, alongside a lack of information on measurement reliability, agreement, and accuracy, could significantly undermine the quality of future studies involving US measurements of masseter muscle thickness. Therefore, the aim of this study is to present a new standardized method for assessing the masseter muscle thickness and to evaluate the reliability, consistency, and accuracy of its US measurements in adults. We also aim to cross-reference the results of masseter muscle thickness measurements obtained via US and CT. We believe this could contribute to the introduction of a unified method for assessing masseter muscle thickness in both scientific research and clinical practice.

## 2. Materials and Methods

### 2.1. Setting and Study Design

US measurements were performed in the Musculoskeletal Elastography and Ultrasonography Laboratory at the Jerzy Kukuczka Academy of Physical Education. CT scans were collected from the Radiology Department at an outpatient clinic. US measurements (thickness) were recorded by two experienced physiotherapists. CT scan measurements were performed by two medical doctors (a radiologist and a dentist) separately. Initially, the CT scan was taken, and then the US was performed within a period of 7 days. The study was authorized by the Bioethics Committee for Scientific Studies at the Academy of Physical Education in Katowice on 21 April 2022 (Decision No. 2a/2022). Procedures and methods were performed in accordance with the relevant guidelines and regulations. All participants gave their signed and informed consent to participate. 

### 2.2. Participants

Participants were recruited at the dental clinic from June to September 2022 from among patients referred for CT to exclude dentogenic infection foci. Participants were eligible for inclusion if they (1) had not received any dental treatment during the study period (between CT and US); (2) had no history of craniofacial trauma, orthodontic treatment, genetic diseases, craniofacial tumors, masticatory organ dysfunction; (3) had full dental arches (excluding teeth 8); (4) had no abnormalities on CT scans; (5) did not exhibit dysfunctions and parafunctions of a craniofacial nature or of the TMJ.

### 2.3. Ultrasound Measurements

An Aixplorer ultrasound scanner (Product Version 12.2.0, Sofware Version 12.2.0.808, Supersonic Imagine, Aix-en-Provence, France) coupled with a linear transducer array (2–10 MHz; SuperLinear 10-2, Vermon, Tours, France) was used to assess masseter muscle thickness. The US scans were collected in supine rest position with the mandible in a relaxed position.

**Figure 1:**
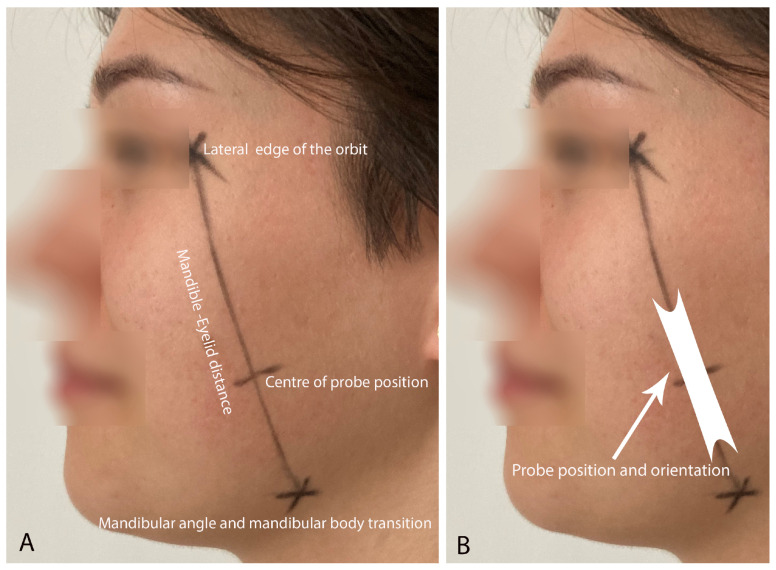
Determination of probe placement (**A**). Final probe position and orientation (**B**).

Prior to the US measurements, for each participant, the precise location of the probe was determined by a dentist and a trained assistant (a physiotherapy student). The precise location of the US probe was based on previous analysis of anatomical preparations and the authors’ own experience. The proposed procedure was as follows (Figure 1A). (a) The distance on the subject’s skin between the lateral edge of the orbit in the pupil line and the point where the mandibular angle transitions into the mandibular body was determined and drawn, called the mandible–eyelid (M-E) distance; (b) the determined M-E distance was measured with a digital caliper in millimeters; (c) the value of 40% of the M-E distance was calculated, and this value was then measured from the transition point of the mandibular angle into the mandibular body; (d) the obtained point indicates the position of the center of the US probe; (e) this center of the US probe was marked. This procedure allows precise and reproducible positioning of the US probe in the frontal and sagittal plane while considering probe rotation (Figure 1B). The center of the probe is placed at the 40% point (the center of probe position) while the entire probe was placed parallel to the M-E distance (Figure 1B). A US image was taken 3 times by each physiotherapist, on both sides of the face, respectively.

### 2.4. Computed Tomography

CT (Device Observer Manufacturer: PNMS, Device Observer Model Name: NeuViz 16, Device Observer Serial Number: N16E100009; Shenyang, China) was performed in the supine position. All subjects were instructed by the dentist to maintain a resting mandibular position during the CT scan procedure. The CT scan was performed with an accuracy of N*T [mm] 0.75 mm. 

### 2.5. Data Analysis

#### 2.5.1. Ultrasound Parameters

**Figure 2:**
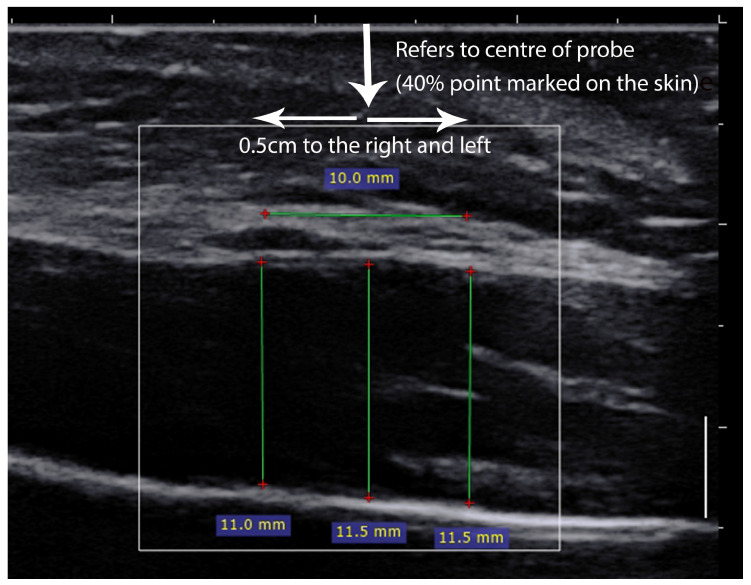
Illustration of ultrasound measurements of masseter muscle.

The US images were saved on an external drive in DICOM format and transferred to a computer where they were further processed using RadiAnt DICOM Viewer (Medixant, Poznań, Poland) to assess masseter muscle thickness. Thickness measurements were taken at 3 locations: (A) in the middle of the picture (this refers to the center of the probe position point); (B) an additional 2 locations 0.5 cm to the left and 0.5 cm to the right from point A, respectively (Figure 2). In further analyses, the average thickness value from these three locations indicated the mean masseter muscle thickness for a given image. 

#### 2.5.2. Computed Tomography

**Figure 3:**
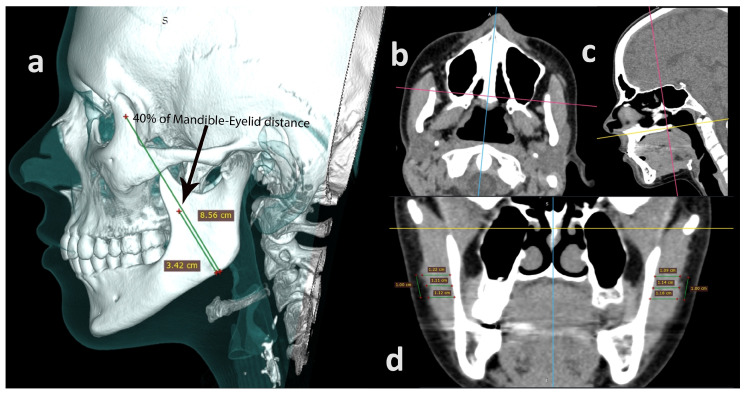
Presentation of masseter muscle thickness measurements by computer tomography. (**a**) 3D VR image; (**b**) horizontal plane; (**c**) sagittal plane; (**d**) frontal plane.

CT scans were saved on an external drive in DICOM format and transferred to a computer where they were further processed using RadiAnt DICOM Viewer (Medixant, Poznań, Poland) to assess masseter muscle thickness and M-E distance. First of all, length measurement of the M-E distance was performed on a 3D Virtual Reality (VR) scan between two referential points (mandible, eyelid) using the first Bone and Skin mode. This procedure involved viewing the patient from a profile perspective. Secondly, the M-E distance was determined by connecting the following points: the lateral edge of the orbit at the pupil line and the point where the mandibular angle transitions into the mandibular body. Furthermore, a point was marked 40% of the distance from the lower end of the segment defining the center of the masseter muscle and the area of the maxillary tubercle (Figure 3a). Afterwards, a soft tissue window was selected and a multiplanar reformated reconstruction (MPR) mode was activated. On the MPR, the image was adjusted according to the anatomical planes as follows: horizontal plane—the point passing between the anterior and posterior nasal spines; (Figure 3b) sagittal plane—the line between the anterior and posterior nasal spike; (Figure 3c) frontal plane—the thickness of the masseter muscle measured at the level of the maxillary cusp (this position was equivalent to 40% of the M-E distance). Finally, two thickness measurements were taken 0.5 cm up and 0.5 cm down from a predetermined point on the external surface of the masseter muscle (Figure 3d) In further analyses, the mean thickness value from these three locations indicated the mean masseter muscle thickness for a given body side. All measurements were taken on the right and left sides of the face. 

### 2.6. Statistical Analyses 

For the calculation of intra- and inter-rater reliability, Intraclass Correlation Coefficient (ICC) type 3,1 and type 2,2 were used, respectively. The ICC was interpreted as follows: 1.00–0.75 (excellent), 0.74–0.60 (moderate), 0.59–0.40 (fair), and below 0.40 (poor reliability) [27]. The agreement was calculated by assessing (1) the standard error of measurement (SEM = SD × √(1 − ICC), with SD representing the standard deviation of the measure; and (2) the results of the Bland and Altman test (BA). The BA test was performed by plotting the difference between the two measurement techniques against their average in order to identify potential systematic errors. The accuracy was calculated as a percent error using the following formula: [(CT measurement − US measurement)/CT measurement] × 100. Additionally, correlation between the two measurement techniques was evaluated using the Pearson correlation coefficient. The Pearson correlation coefficient was graded as r < 0.2 for very weak, 0.20–0.39 for weak, 0.40–0.59 for moderate, 0.60–0.79 for strong, and ≥0.8 for very strong correlation [14].

## 3. Results

### 3.1. Participants

Finally, the study included nine participants (three men and six women; mean age: 25.4 years; mean body mass: 63.5 kg; mean body height: 172.8; BMI: 21.4 kg/m^2^) who met the inclusion criteria. 

### 3.2. Masseter Muscle Thickness (Reliability and Agreement)

The masseter muscle thickness measured by CT over a seven-day interval received excellent intra-rater reliability with a coefficient of variation (CV) below 1.5%. The BA test revealed systematic error in the masseter muscle measured on the right side by a radiologist, meaning that during the second measurement, this muscle was slightly thinner (bias 0.21 mm) compared to the first measurement. The inter-rater reliability for the first CT measurement of the masseter muscle was excellent, with a CV below 1.5%. The BA test showed no systematic error (Table 1).

The masseter muscle thickness measured by US received excellent intra-session reliability (with a 15 min interval between measurements). The corresponding CV values varied from 2 to 4% for the first assessor, whereas the CV value for the second assessor was below 1%. The inter-rater reliability for the first US measurements performed over a seven-day interval were also excellent, with corresponding CV below 3%. The BA test form US measurements showed no systematic error (Table 1). 

### 3.3. Mandible–Eyelid Distance

**Figure 4:**
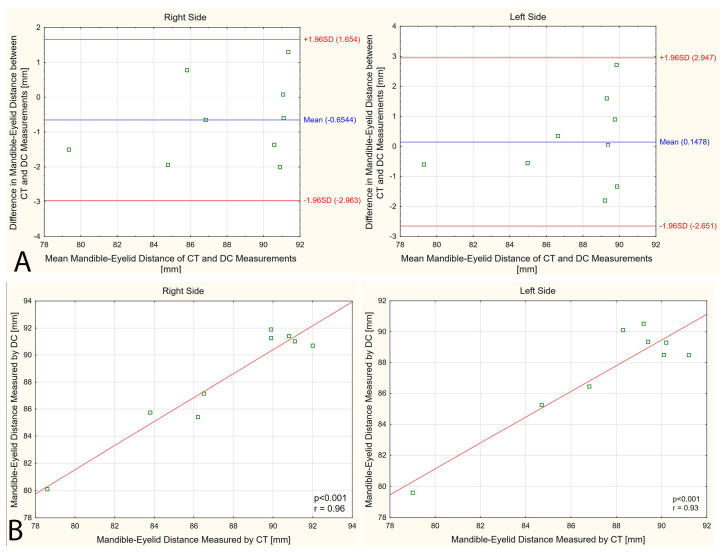
Analysis between computer tomography (CT) and digital caliper (DC) measurements of the mandible–eyelid distance: (**A**) Bland–Altman plot; (**B**) Pearson correlation.

The mean M-E distance measured by CT was 87.6 mm (4.34) and 87.7 mm (3.80) on the right and left sides, respectively. The mean M-E distance measured by digital caliper was 88.3 mm (4.00) and 87.5 mm (3.41) on the right and left sides, respectively. Mean percent error in the M-E distance measurement between CT and the digital caliper was below 1.4% regardless of body side. The exact percent error varied from 0.05% to 3% within the examined population. The Bland–Altman analysis has shown that the bias for the M-E distance measurement by CT and digital caliper was close to zero with no systematic error (Figure 4A). A significant linear relationship was shown between the CT and digital caliper measurements on both body sides (*p* < 0.001, r ≥ 0.93, Figure 4B).

### 3.4. Masseter Muscle Thickness (CT versus US)

**Figure 5:**
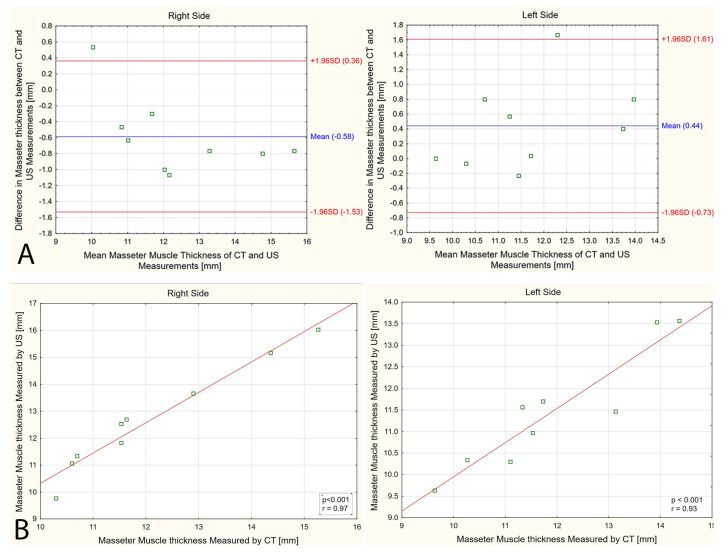
Analysis between computer tomography (CT) and ultrasound (US) measurements of the masseter muscle: (**A**) Bland–Altman plot; (**B**) Pearson correlation.

The mean masseter muscle thickness measured by CT was 12.1 mm (1.74) and 11.9 mm (1.61) on the right and left sides, respectively. In turn, the mean masseter muscle thickness measured by US was 12.7 mm (2.00) and 11.5 mm (1.37) on the right and left sides, respectively. Mean percent error in masseter muscle thickness measurement between CT and US was below 6% regardless of body side. The exact percent error varied from 0% to 12.7% within the examined population. The Bland–Altman analysis has shown that the mean bias for the masseter muscle thickness measurement by CT and US was 0.4–0.6 mm, with systematic error detected on the right side (Figure 5A). A strong linear relationship was found between the CT and US measurements on both body sides (*p* < 0.001, r ≥ 0.93, Figure 5B).

## 4. Discussion

The purpose of this study was to evaluate the reliability, agreement, and accuracy of masseter muscle thickness measurements. This study was undertaken due to the lack of a detailed and validated method for assessing masseter muscle thickness using US. Such validation is necessary to ensure the quality of studies and measurements in future research involving US measurement of masseter muscle thickness, as the masseter muscle is linked to various craniomandibular dysfunctions [1,2,3] and systemic diseases [8,9,10,11,12,13,14,15,16,17,18]. Taking this into account, we have demonstrated in the present study that the proposed method for measuring masseter muscle thickness using US achieved excellent reliability, yielding results similar to those obtained from CT images in healthy adults. Thus, the results obtained allow us to claim that the developed method of assessing the thickness of the masseter muscle using US can be implemented and verified on patients with some craniofacial conditions. 

The systematic review by Blicharz et al. [26] presented various scientific reports that describe different methods for measuring and assessing masseter muscle thickness using US. However, as the authors concluded, none of these procedures have been fully verified for reliability and accuracy. To the best of our knowledge, we have found only a few different methods in the literature for examining the masseter muscle with US [7,18,21,23,24,25,26,28]. Volk et al. [25] positioned the US transducer perpendicular to the skin surface and transverse to the segment of the zygomatic bone arch and mandibular angle. Satiroglu et al. [24] positioned the US transducer on the skin surface in the middle of the mandibular branch, without taking into consideration the actual middle (center) position. Strini et al. [7] positioned the US transducer perpendicular to the course of the muscle fibers of the masseter muscle in the midsection between the zygomatic arch and the angle of the mandible based solely on palpation. Park et al. [23], in contrast to the mentioned works, has possibly presented the most reproducible methodology for examining the masseter muscle thickness by US. Park et al. [23] positioned the ultrasound probe perpendicular to the course of the masseter muscle at a level that coincided with the occlusal plane of the studied subjects. However, it seems that the intraoral line (occlusal plane) and the extraoral line (probe position) will not match in each subject. Ispir et al. [28] positioned the ultrasound probe perpendicular to the masseter muscle in the anteroposterior direction, and the measurement was taken at the widest point in the transverse section. In turn, González-Fernández M. et al. [18] positioned the transducer perpendicular to the external edge of the muscle (between the intertragic fissure and the oral commissure, parallel to the Frankfort plane). 

Thus, taking all mentioned studies into consideration [7,18,23,24,25,26,28], it should be pointed out that the location of the US probe placement was not precise, and the examiners had some freedom in probe position. Therefore, in the present study, it was decided to determine the position of the US transducer with the greatest possible precision by drawing lines on the subject’s skin. Certainly, this method extends the duration of the examination, but enables highly reproducible US probe placement (imposes a precise probe alignment in all directions). This approach has not been used in other scientific studies on the US measurement of the masseter muscle. Our novel method of examining the masseter muscle with US could be highly relevant, especially for repeated measurements of muscle thickness at intervals in clinical trials. In addition, in the proposed method, the probe is positioned along the muscle fibers, which may be particularly useful in shear wave elastography of the masseter muscle. 

In scientific research, an important aspect is the reliability of the methods used to evaluate tissues (US of masseter muscle thickness in this study). In this regard, the methods proposed by Strini et al. [7] and Volk et al. [25] were not validated. Park et al. [23] only presented reliability results for one and two repeated measurements, where ICC results of 0.7–0.9 were obtained. In none of the mentioned works were accuracy and agreement assessed. In our study, we presented an assessment of the reliability, agreement, and accuracy of US measurements of the masseter muscle and compared the US results with CT results. The results have shown that in the US evaluation of the masseter muscle, the ICC value exceeds 0.8, there were no systematic errors, and the results were highly correlated with CT. 

### Implications and Limitations

In this present study, we demonstrated that the emerging new method of masseter muscle US assessment can be validated on clinical material and then used in clinical studies to evaluate changes in masseter muscle thickness, or in observational studies of masseter muscle thickness variability in different populations. It might also be possible to study the effect of additional factors, i.e., pharmacotherapy, physiotherapy, and surgical treatment, on the thickness of the masseter muscle by using such a method. 

The present study has several limitations. First of all, the study included only nine healthy adults. Thus, the results should be considered as preliminary and cannot be extrapolated to children, adolescents, or patients with specific conditions. Second, measurements were collected in the supine rest position, whereas body position can affect the masseter muscle measurements. Furthermore, we do not know if this proposed method will be suitable for assessing the masseter muscle in contraction. Lastly, reliability of the proposed method is still affected by the palpation needed while determining the proper probe positioning. Hence, the person drawing lines on the face in our study received 10 h of hands-on training. 

## 5. Conclusions

The proposed method of masseter muscle thickness measurement using US has demonstrated excellent reliability with similar results to those obtained from CT images in a healthy adult population. The results allow us to conclude that the developed method of assessing the thickness of the masseter muscle using US can be implemented and verified on patients with some craniofacial conditions. Therefore, we recommend investigating this method in further studies, where precise assessment of the masseter muscle by US is expected. 

## Figures and Tables

**Table 1:** Reliability and validity of masseter muscle thickness measurement by computer tomography and ultrasonography.

		Masseter Muscle Thickness	
		Right Side	Left Side
Computer Tomography			
Intra-rater reliability Radiologist (seven-day interval)	ICC_3.1_ (95% CI) ^1^	0.99 (0.97–0.99)	0.98 (0.93–0.99)
	SEM (mm)	0.17	0.22
	CV (%)	1.22	0.81
	Bias ^3^ (mm)	−0.21 ^2^	−0.14
Intra-rater reliability Dentist (seven-day interval)	ICC_3.1_ (95% CI) ^1^	0.98 (0.91–0.99)	0.97 (0.89–0.99)
	SEM (mm)	0.27	0.26
	CV (%)	0.28	0.40
	Bias ^3^ (mm)	0.05	0.07
Inter-rater reliability	ICC_2.1_ (95% CI) ^1^	0.88 (0.60–0.97)	0.98 (0.92–0.99)
	SEM (mm)	0.63	0.22
	CV (%)	1.33	0.89
	Bias ^3^ (mm)	−0.23	−0.22
Ultrasonography			
Intra-session reliability First physiotherapist	ICC_3.1_ (95% CI) ^1^	0.85 (0.47–0.96)	0.87 (0.53–0.97)
	SEM (mm)	0.68	0.56
	CV (%)	1.97	3.59
	Bias ^3^ (mm)	−0.36	−0.60
Intra-session reliability Second physiotherapist	ICC_3.1_ (95% CI) ^1^	0.98 (0.93–0.97)	0.94 (0.76–0.99)
	SEM (mm)	0.25	0.41
	CV (%)	0.30	0.55
	Bias ^3^ (mm)	0.05	0.09
Inter-rater reliability	ICC_2.1_ (95% CI) ^1^	0.92 (0.71–0.98)	0.87 (0.44–0.97)
	SEM (mm)	0.54	0.53
	CV (%)	1.48	2.93
	Bias ^3^ (mm)	0.26	−0.48

CV—coefficient of variation; ICC—intraclass correlation coefficient; SEM—standard error of the mean; ^1^ the 95% confidence interval; ^2^ systematic error as the line of equality is not in the 95% confidence interval; ^3^ Bland–Altman Test.

## Data Availability

The raw data supporting the conclusions of this article will be made available by the authors on request.
